# ‘Learning and growing together’: exploring consumer partnerships in a PhD, an ethnographic study

**DOI:** 10.1186/s40900-023-00417-6

**Published:** 2023-03-15

**Authors:** Ruth Cox, Matthew Molineux, Melissa Kendall, Bernadette Tanner, Elizabeth Miller

**Affiliations:** 1 Occupational Therapy Department, Queen Elizabeth II Jubilee Hospital, Corner Kessels and Troughton Roads, Coopers Plains, QLD 4108 Australia; 2 Discipline of Occupational Therapy, School of Health Sciences and Social Work, Griffith University, Queensland, Australia; 3 Acquired Brain Injury Outreach Service and Transitional Rehabilitation Program, Princess Alexandra Hospital, Buranda, QLD Australia; 4 School of Health Sciences and Social Work, Griffith University, Meadowbrook, QLD Australia; 5 Consumer Co-Researcher C/O Occupational Therapy Department, Queen Elizabeth II Jubilee Hospital, Coopers Plains, QLD Australia

**Keywords:** Consumer and community involvement, Patient and public involvement, Co-production, Epistemic justice, Doctoral research, Qualitative

## Abstract

**Background:**

Consumer and community involvement (CCI) in health research is increasingly recognised as best practice and is closely linked with calls for epistemic justice and more transparent university collaborations with consumers. Given doctoral candidates play a key role in the future of co-production, examination of consumer partnerships in PhDs is important. This study aimed to describe and evaluate consumer partnerships in a PhD from the perspective of the consumer co-researchers, the PhD candidate, and the academic supervisors including optimal approaches, impacts, and benefits and challenges.

**Methods:**

This prospective, co-produced ethnographic study was conducted over 33 months. Data collection included field notes, a monthly online log of partnership experiences and time spent, interviews or a focus group every six months, and a PhD student reflexive diary. Qualitative data were analysed using reflexive thematic analysis.

**Results:**

The student, two academics, and four consumer co-researchers were involved. A mean of 11.10 h per month were spent on CCI. The student spent the most time (mean 15.86 h per month). Preparation for dissemination of findings was the most frequent partnership activity. The two overarching themes emphasised that a PhD promotes a rich partnership ethos with the student at the centre and that the partnership was a worthwhile but challenging process. The four sub-themes highlighted that developing a collegial and supportive environment with regular meetings combined with a multi-faceted and responsive co-learning approach were core to success. Additionally, there were benefits for individuals, research processes and outcomes, and for driving change in consumer-academic research partnerships. Recruiting to and forming the partnership, maintaining the collaboration through inevitable changes and challenges, and an ethical and supportive closure of the research team were critical.

**Conclusions:**

This longitudinal ethnographic study demonstrated that doctoral research can create a rich ethos for research and knowledge co-production which evolved over time. Equalising power dynamics through relationship building and co-learning was critical. Additionally, a focus on supportively ending the partnership was essential, and CCI may reduce PhD student isolation and procrastination. Enhanced university incentivisation of co-production in health research is recommended to address gaps in consumer remuneration and student support.

**Supplementary Information:**

The online version contains supplementary material available at 10.1186/s40900-023-00417-6.

## Plain Language summary

Partnering with people who use health services, their families, and the community (consumers) to do research is ethically the right thing to do and leads to better research. Unfortunately, it often does not happen, and researchers are not sure how to do it. To change this, we want researchers to partner with consumers early in their research career, when they are doing a PhD. However, there is limited evidence about this topic. This study described and evaluated a partnership from the viewpoints of the PhD student, the university supervisors (academics), and the consumer co-researchers over 33 months. We found that the whole team learned and grew together, and that the longer-term learning environment of a PhD helped foster a more equal partnership. The partnership was enjoyable and provided many benefits to those involved and to the studies. Much time was spent sharing research in ways that were better for consumers such as social media, newsletters, and presentations. At the start of the partnership, it was important to set up the team for success and there were many challenges along the way which needed flexibility. It was important to prepare for the ending of the PhD so that everyone felt supported to move on. Two big challenges were that the university did not have resources such as templates or courses to help the student learn about partnerships and did not have dedicated funding to pay the consumers for their time.

**Supplementary Information:**

The online version contains supplementary material available at 10.1186/s40900-023-00417-6.

## Background

Health research which is conducted ‘with’ and ‘by’ consumers rather than ‘for’ or ‘to’ them is now considered best practice [1, 2]. The term consumer includes health service users/patients, carers/families, and communities/the public [3]. Consumer and Community Involvement (CCI) is the terminology used in Australia to describe the incorporation of the experiential knowledge of consumers into health research beyond being a study participant [3]. Greenhalgh et al. [4] described the three main perspectives that support CCI in research. Firstly, the normative ethos emphasises consumers’ right to prioritise and influence research which is about them, and researchers’ obligations to address power imbalances and reduce health inequities. Secondly, the consequentialist approach focusses on efficiency, emphasising that CCI brings consumers’ lived experience to improve research methodologies and translation. Thirdly, political and practical arguments reinforce formation of researcher-consumer alliances to increase research accountability and transparency, and to attract resources [4]. CCI should occur across the research cycle and be tailored to each unique research project and activity [5, 6].

Despite CCI in health research being public policy and mandated by many funding bodies, it is not commonplace, and is frequently tokenistic [7, 8]. Co-production is commonly discussed in the CCI literature and entails researchers and consumers working as genuine partners, sharing power, and responsibility in research, from project inception to conclusion [9]. In co-production, each partner brings their complementary and mutually beneficial expertise [10]. It includes a more reciprocal, evolving, and reflective way of working together as a team [11] and a kinder approach to building ongoing relationships that extend beyond a particular study [8].

The core principles of co-production are compatible with contemporary epistemic justice, where the knowledge of all social groups is valued [7], and knowledge mobilization where end users are central to translating research evidence into improvements in healthcare [12]. In these paradigms new partnerships are formed outside traditional academic institutions to grow a knowledge-based society [13, 14]. The civic role of universities in delivering value to communities and universities as ‘just anchor’ institutions which deliver social, economic, and epistemic justice benefits through co-production with, and empowerment of, the public [14] are also aligned with the CCI ethos [8]. Limited uptake of CCI in research may be due to a lack of academic knowledge and inadequate university support [8]. In particular, doctoral researchers have strict time and financial boundaries and so may be discouraged from adding CCI [15]. With global expectations that universities increase their number of doctoral students [16], and the potential for early career researchers to foster a research culture that values CCI [13, 17, 18], it is highly appropriate to investigate CCI in doctoral research.

The limited papers which examine CCI in PhDs indicate that consumer engagement positively contributes to recruitment, methodological rigour, data analysis, interpretation, and dissemination of findings [17, 18]. The importance of flexibility in responding to consumer learning and support needs and in building relationships is emphasised [17, 19]. Challenges include unclear ethical guidance, time limitations, and difficulty in ensuring adequate consumer support [18]. Whilst these papers provide powerful narratives and recommendations for success, they all entail retrospective reflections from the perspectives of doctoral candidate(s) [18, 20], or both the candidate(s) and consumers [15, 17, 19]. None of the papers have presented the supervisor perspective. Thus, there is a need for additional formal, prospective evaluation of co-production of doctoral research with consumers from the perspectives of all partners. Hence, the aim of this study was to describe and evaluate consumer partnerships in a program of doctoral research from the perspective of the consumer co-researchers, the PhD candidate (student), and the academic supervisors (academics).

## Methods

### Research questions

To meet the study aim, the research questions were:

- What are the optimal approaches to consumer partnerships in a PhD?
- What are the impacts of consumer engagement strategies on the processes and outcomes of a PhD from the perspective of the consumer co-researchers, student, and academics?
- What are the benefits and challenges of consumer partnerships in a PhD from the perspective of the consumer co-researchers, student, and academics?

### Overview of the PhD studies

**Fig. 1:**
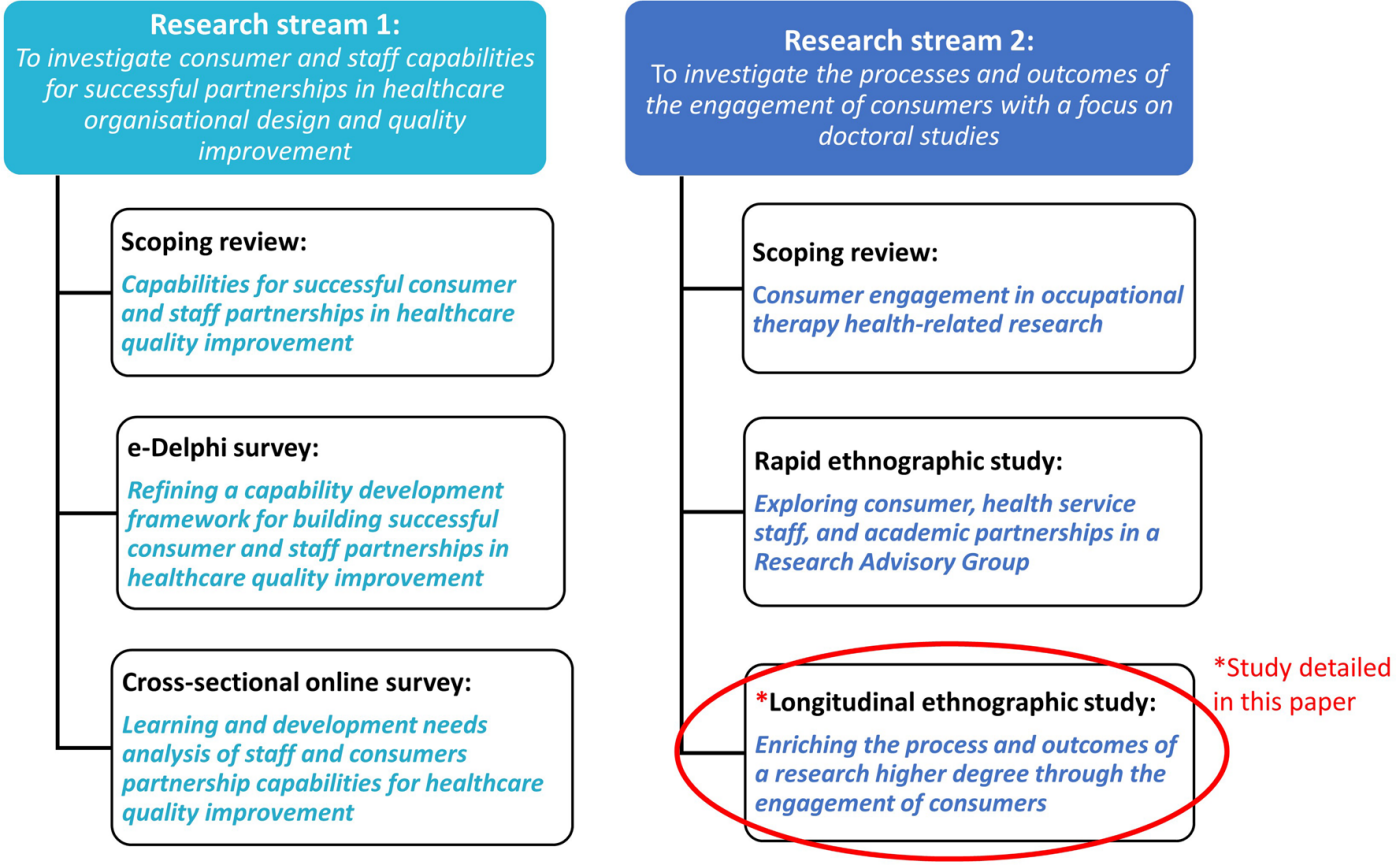
The six PhD studies across two research streams

The PhD research examined in this paper included six studies (including this one) across two streams of research which were highly relevant to consumers. Stream 1 focussed on development of a capability framework for successful staff and consumer partnerships in healthcare quality improvement. Stream 2 investigated the processes and outcomes of the engagement of consumers in research with a focus on doctoral studies. Figure 1 includes the six studies and research streams. The ethnographic study presented here is circled in Fig. 1. It focussed on co-production with four consumer co-researchers across the PhD including when a research advisory group (RAG) with consumers and staff from diverse backgrounds including First Nations, culturally and linguistically diverse, and disability, was incorporated into the design of the eDelphi study.

### Study design

An ethnographic study design was appropriate to holistically unveil the social and cultural processes inherent in the co-production group setting and the significance and meaning that was attributed to behaviours, interactions, and practices [21]. An insider perspective from the research team inclusive of the student, academic supervisors, and consumer co-researchers was gained over a 33-month period, using multiple and systematic methods. Data gathered provided a comprehensive picture of group identity, beliefs, values, actions, and power dynamics [22] which was aligned with the research team’s philosophical commitment to CCI. The GRIPP2-SF (Guidance for Reporting Involvement of Patients and the Public‐Short Format) checklist [2] guided reporting of CCI for this study. Additional file 1 includes a response to each item.

### Recruitment

An expression of interest was emailed to approximately 125 consumer partners and volunteers of a large Australian metropolitan Hospital and Health Service (HHS). Interested consumers were invited to discuss the study and role expectations with the student. Supportive and relaxed interviews were then conducted by the student and at least one academic supervisor. See Box 1 for details of the selection criteria which were shared with the consumers and guided the interviews. The student and academics provided informed consent, but formal recruitment was not necessary.

**Box 1:** Consumer recruitment selection criteria

• Ability to articulate a consumer perspective and respect and appreciate different perspectives expressed within the research team
• Ability to commit sufficient time to participate fully in the research including two hours per month meeting preparation and two hours per month meeting attendance
• Confident to discuss ideas in a group setting
• Ability to read and understand written research information, or able to seek assistance to understand the information
• Ability to access email and a computer, or able to regularly attend the hospital to receive and discuss documents
• Ability to add to the diversity of viewpoints of the research team by providing an individual perspective of a patient or family member/carer of a patient from one or more of the following backgrounds:
• 65 years old or more
• Non-english speaking or overseas culture
• Aboriginal and/or Torres Strait Islander
• Disability

### Study processes

Recruited consumer co-researchers undertook mandatory HHS induction including code of conduct and confidentiality training. The whole research team attended monthly meetings of two hours duration with ad hoc or longer meetings, as agreed. Meetings were face to face at a hospital until COVID-19 restrictions necessitated online videoconferences. Pre-reading was prioritised (essential, useful, of interest) and sent one week in advance. Meeting agendas included a review of the previous meeting (written summary); check-in about ethical issues; review of the agenda, decisions, and achievements; addition of relevant emerging agenda items with time for discussion and learning; check-out regarding ethical issues and meeting processes; and planning for the next meeting. Time for informal relationship building was also prioritised. Key papers regarding best practice in research partnerships [23], ethical issues in CCI [24], and the International Association of Public Participation (IAP2) spectrum [25] were introduced early and revisited regularly to inform reflexive discussion.

### Data collection

**Fig. 2:**
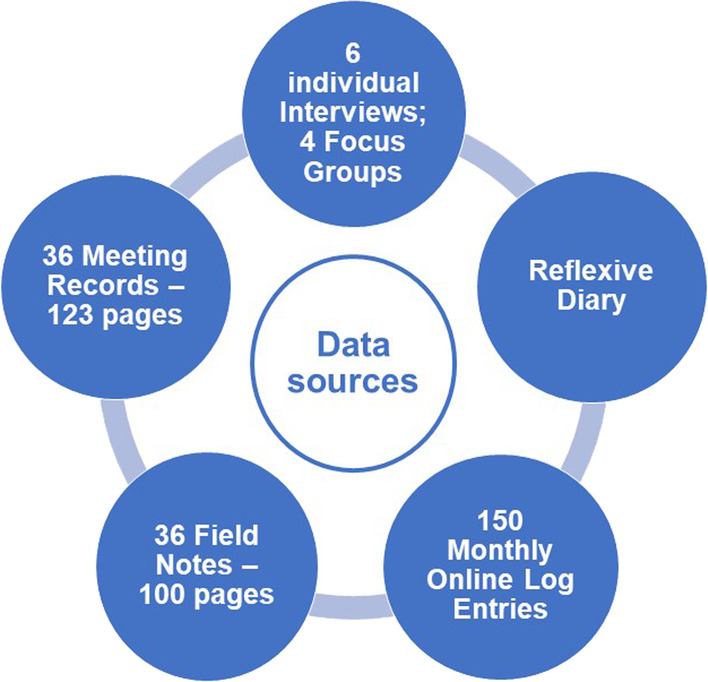
Data sources

Consistent with ethnography [21, 26], multiple data collection strategies were used. See Fig. 2 for a summary. A monthly online log (Log) regarding time spent on CCI for the PhD studies, research activities engaged in (from a list), reflections on enjoyment and satisfaction, time spent, and reflections on engagement in CCI in external research was completed by all team members. Additionally, either an in-depth, semi-structured interview (IV) or focus group (FG) was conducted every six months with the method chosen as a team. Interviews were led by the student. Focus groups were facilitated by an external expert in qualitative research methods. The interview and focus group guide explored what went well and what did not go well in the research partnership during the past six months; degree of consumer influence; benefits and challenges; ethical issues; and learning and support. The student also documented and shared meeting summaries and kept comprehensive field notes (FN) at all meetings. The field notes including direct quotes, observations of verbal and non-verbal reactions and interactions, and personal reflections. Additionally, she maintained a reflexive diary to facilitate self-awareness and reflexivity about beliefs, assumptions, and reactions [26].

### Data analysis

Quantitative data were analysed using descriptive statistics. The team used a six-stage process of reflexive thematic analysis [27] for qualitative data analysis. This was highly suitable for exploration of the meaning behind data, consistent with ethnography [26]. The team had effectively used reflexive thematic analysis for two previous studies [28, 29] and a conference presentation. Consistent with Braun and Clarke’s (2021) recommendations, the team undertook many reflexive discussions regarding paradigmatic, epistemological, and ontological assumptions which informed analysis. The team’s commitment to authentic CCI and social justice, individuals’ healthcare experiences, and cultural, disciplinary, and socioeconomic backgrounds influenced data interpretation.

To maximise trustworthiness [26], the student and academics independently coded one example of each data type and compared coding. The minimal discrepancies were negotiated, and coding adapted. The student then coded all remaining data using coding software. Early preliminary themes were developed by the student and discussed with the academics who had access to all data. Small changes were made. Given the large data set, the consumers agreed that preliminary themes with indicative data, rather than the whole data set, would be reviewed as a team. Consumer collaboration on data analysis occurred over three months and four meetings. Themes were gradually refined, defined, and named. Other trustworthiness strategies [26] included multiple data sources, a memo trail, review of research meeting summaries, and ongoing team reflection regarding whether themes captured experiences.

## Findings

### Who was involved?

Three consumers were initially recruited. One consumer withdrew in month five. Recruitment was repeated, and one new consumer joined the research team in month seven but withdrew in month 10. A third recruitment round was not attempted due to the time required, the reported impacts of COVID-19 on the well-being of consumers, the inability to meet face to face, and the high degree of influence of the two consumers who remained on the team. The seven people involved in the study included the student, two academics, and four consumer co-researchers (the research team). Table 1 outlines the personal characteristics of the research team. Individual characteristics are not reported against roles to protect anonymity.

**Table 1:** Characteristics of the research team

Characteristics	Details
Age and gender	• Average age at recruitment 55.71 years (range 48–69) • 2 males (28.6%)
Work and voluntary roles	• One hospital and community occupational therapy director and consumer partnering lead • One university professor and occupational therapy department head • One senior researcher in community rehabilitation services with an associate professor appointment and psychology degree • One retired pathology scientist and hospital volunteer • One retired primary care policy implementation manager and consumer advisor at national, state, and local levels • One retired university finance director and hospital volunteer • One semi-retired small business owner and hospital volunteer
Health and consumer lived experiences	• One person with a disabling autoimmune condition • One person with a neurological condition that affected talking, eating, and drinking • One primary carer of an older person with a degenerative neurological condition • Three family members of elderly parents and relatives with multiple hospital admissions and primary care support needs • One sibling of a person living with a disability following severe brain injury • One person identifying as LGBTIQ + • One person with immigrant parents who did not speak English • One close friend of a person who had undergone long periods of involuntary admission to mental health inpatient units

### Quantitative data

Thirty-seven research team meetings occurred during the 33 months of data collection. Meetings were more frequent during intense periods of data analysis and interpretation, and for consumer orientation or learning support. Figure 2 provides details of the data collected. Of note, the six interviews included one withdrawal interview. The second consumer who withdrew was interviewed at six-months then was lost to follow-up. One academic ceased log data entry at 16 months due to time pressures. Their time data were excluded from analysis. The rest of the research team spent a mean of 11.10 h per month on CCI associated with the PhD. As expected, the student spent the most time per month (mean 15.86 h). The consumers spent more than double the anticipated four hours per month (mean 8.75 h) which was similar to the time spent by the academic (mean 8.70 h).

**Fig. 3:**
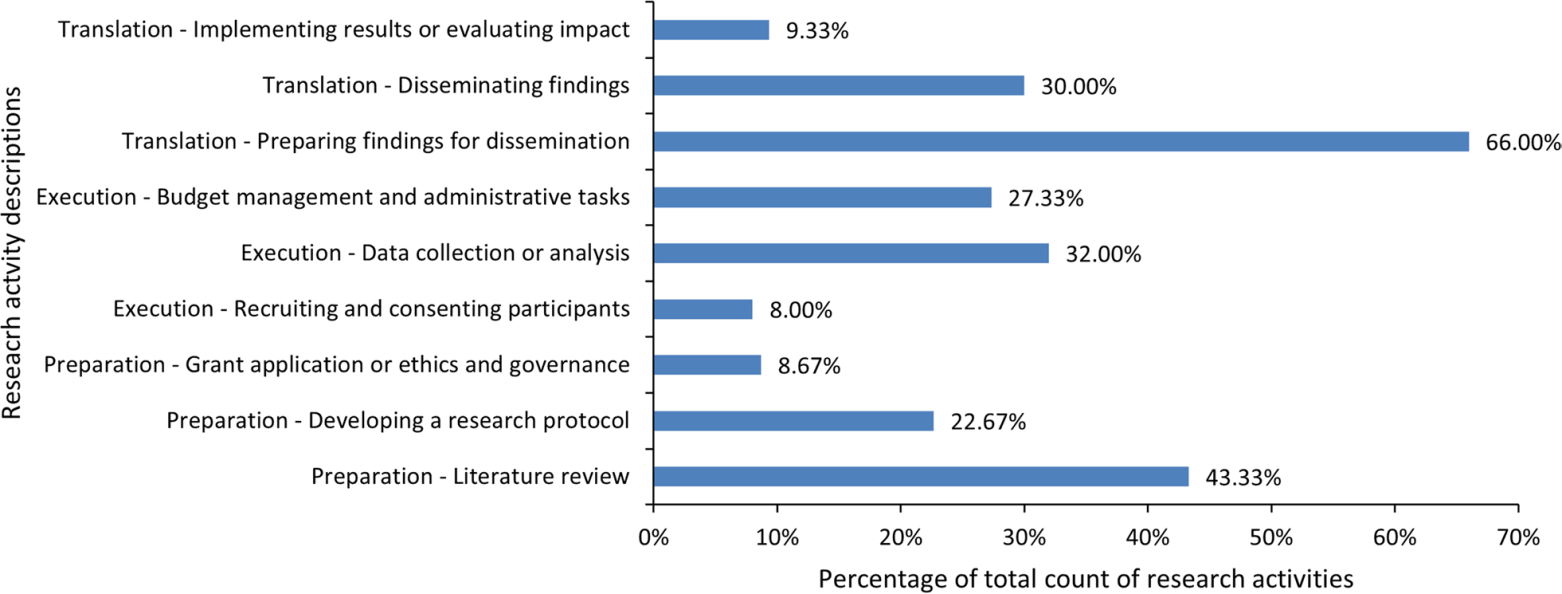
Reported research team consumer and community involvement (CCI) activities in past month across the research cycle

The research team collaborated in a variety of CCI activities across the research cycle as indicated in Fig. 3. Preparation of findings for dissemination was the most frequent activity. Four co-authored peer-reviewed publications resulted from the partnership, excluding this one [28–31]. Additionally, there were seven local, two national, and four international co-presentations and posters, and six consumer-focused newsletter pieces published plus many tweets.

### Qualitative data

Reflexive thematic analysis resulted in two overarching themes and four sub-themes which elaborate on both of the overarching themes. Gender neutral pseudonyms, role (student, academic, consumer), and data source are reported for direct quotes where data were not anonymous.

### Overarching theme: learning and growing together

The core philosophy of doctoral research, with its focus on student development and leadership with academic supervisor support over a prolonged period, enabled the rich partnership. After a year of collaboration, Alex reflected on how far the group had progressed, *“…we're learning and growing together. …”* (Alex- Consumer- FG-12mths) The student was at the centre with strong altruism expressed by the consumers, *“…to see you walk across the stage and have your PhD confirmed … I’ll think, I've had a bit of part to play in that.”* (Jerry-Consumer-IV-6mths) The position of the student as a learner set the scene for balancing power dynamics, inclusion, and teamwork. However, the PhD required student leadership as described by Alex, *“We can be empowered by the processes… but then we're not directing this piece of work. You are [student]… that's a good thing for us to recognise… I'm glad I don't have that responsibility.”* (Alex-Consumer-IV-6mths) This was reinforced by an academic who commented on the parameters of a PhD and, *“…expectations that we all have to meet… particular things about [student’s name] driving, [student’s name] originality, and all of that sort of stuff….*” (Sam-Academic-FG30-mths) However, there were examples of consumer leadership including theme naming for two qualitative studies, providing introductions and generating contacts for the RAG recruitment, and sharing dissemination materials such as newsletters and publications with consumer networks.

The consumers expressed gratitude and admiration regarding the academic supervisors’ unique contributions and continuous presence, *“…[the] supervisors are just front and centre and so inclusive of us….”* (Chris-Consumer-FG-30mths) The 33-month partnership across multiple studies, which is somewhat unique to a PhD, enabled relationships, capabilities, and achievements to grow. Additionally, university milestones provided a check point for team progress and achievements with the academic rigour of the formal evaluation facilitating reflection on group culture and partnership strategies. This fostered transparency, reflexivity, and many opportunities for iterative improvements of group processes. The student also commented, *“…having the two consumers at my PhD confirmation seminar. I felt really supported and valued.”* (Regi-Student-Log-12mths)

### Overarching theme: a worthwhile but challenging process

Throughout the partnership there were uncertainties, challenges, and boundaries which ebbed and flowed over time. However, with team flexibility, and an ethical approach, whilst not easy, issues were addressed, resulting in an overall positive experience. An academic highlighted the importance of advocacy and the slow nature of change, *“We need to chip away at the edges. Even if we break down one barrier in the system it’s a victory.”* (Pat-Academic-FN-Mtg3-4mths) The burden mainly fell to the student with the consumers feeling protected and less emotional about challenges. Chris said, *“…it's [student’s name] project, we are along to support her… I don't take it on personally as my frustration….”* (Chris-Consumer-FG30mths) Additionally, the student juggled the consumers’ desire for quick action with research timeframes with Alex remarking*, “…I wish the wins would be faster rather than slower (laughter), and not just chips, but big rocks of change… avalanches….”* (Alex-Consumer-FG30mths) The student creatively supported the consumers and accommodated preferences, such as pre-recording co-presentations, printing documents, debriefing after meetings, and being a sounding board when dilemmas arose. Whilst extremely important, this added a time burden and complexity to the PhD but with practice became business as usual and more efficient as templates and processes were developed and repeated. The team discussed that not all students may have the capabilities to effectively lead such a rich partnership with the associated uncertainties and complexities. Additionally, sustainability of academic supervisor time was queried.

Withdrawal of two consumers due to time pressures and personal circumstances required student time and effort for recruitment and orientation. Additionally, attracting only well-educated consumers potentially perpetuated societal inequities which did not sit comfortably with the team. However, being able to read and comprehend at a high level was agreed to be necessary for meaningful collaboration. Additionally, the need for stronger cultural and ethnic diversity was addressed for a main PhD (eDelphi) study through development of a short-term RAG. Whilst the RAG successfully provided diverse viewpoints, it created a large student workload and the team felt that the depth of collaboration gained in a smaller group was not achieved. Pushing boundaries such as development of a suitable organisational-consumer research agreement and negotiating consumer co-presenter complimentary conference attendance was relished due to the team advocacy focus. However, this required sustained energy and passion, especially for the student as it seemed that a new unanticipated challenge emerged with each study. An academic commented, *“…we're at the clash of these things… challenging the status quo… with the journal, with the conference, and behind that is … no, this is our special, professional space.”* (Sam-Academic- FG-12mths) Lack of university infrastructure for CCI in research and no budget or incentive for consumer remuneration were also challenging. The two long-term consumer co-researchers only received a small stipend of AUD$950 each from two funding sources associated with the health service.

### Sub-Theme: a relaxed, cooperative, and friendly environment

Over the 33 months**,** the small research team developed collegial and supportive relationships which strengthened and deepened and were focussed on a common goal. Even as early as six months, Chris, a consumer, described the group atmosphere as *“Relaxed and cooperative and friendly….”* (Chris-Consumer-IV-6mths) She felt similarly at two years, *“I love our little group, and I feel really involved and engaged in all the relationships.”* (Chris-Consumer-FG-24mths) Getting to know each other and reconnecting at meetings including holiday banter, humour, and debriefing about COVID-19 and personal challenges built and maintained a warm and enjoyable environment. Making a conscious effort moved to being relaxed and natural. Sam, an academic, discussed the change in mindset, “*…power is different and you’ve… got to be aware of supporting someone but then not being patronising… normally if you're working with a consumer as a health professional… you're there for them. Whereas this, we're there for each other.”* (Sam-Academic-IV-6mths)

Monthly meetings required considerable preparation but created momentum and accountability for all. The group size of 5–6 people was also beneficial and contrasted with the eDelphi RAG of 12 which *“…lacks the warmth and ease of the research team.”* (Academic-Log-24mths) The formal meeting structure also supported equal contributions, *“…you have given us an agenda… a PowerPoint. I have a systematic way… to know what… I should be focusing on so that I can be a part of the meeting.”* (Ashley-Consumer-IV-6mths) The student ensured meaningful consumer collaboration by facilitating their engagement in practical work such as refining research questions; accuracy checking of literature mapping; collating, refining, and naming themes; choosing layouts of graphs and tables; generating discussion points for manuscripts; and proof reading. This required considerable planning but reinforced peer relationships and became easier over time as preferences became known and skills developed. For example, aspects of qualitative data analysis were conducted on large printouts attached to walls to accommodate consumer preferences in conjunction with social distancing during the first year of the partnership. However, by the third year, a similar data analysis was conducted via videoconference with screen sharing.

Working with a consumer who had extensive partnering experience and networks, and a consumer who was new to partnering broadened the perspectives included whilst also expanding networks for dissemination and translation of findings. Consumers’ contributions to rich discussion required bravery and a high level of mutual acceptance, even in disagreement. Additionally, a proactive approach to ethical issues with reflective discussion about relationships, stressors, boundaries, confidentiality, and consent built into the agenda ensured that, *“…everybody is thinking about it…which hopefully then would uncover if there was an ethical issue brewing… so it could be addressed right before it festered into something ugly.”* (Pat-Academic-IV-6mths)

### Sub-theme: natural, comfortable, and applied learning

Contextualised and responsive co-learning was critical. Learning strategies were varied and prepared by the student, including pre-reading, online videos, presentations, learning by doing, and considerable reflexive and reflective discussion. The consumers embraced their learning journey expending considerable time and mental energy re-reading information several times. Jerry commented, *“So, I'm down here on the learning curve, but I expect it to ramp up.”* (Jerry-Consumer-IV-6mths) Student flexibility was paramount as the topics the consumers wanted to explore in-depth, or which emerged as important, such as predatory journals, thesis requirements, and positionality, were not always predictable. Chris, a consumer, talked about the learning approaches feeling *“…natural, comfortable, and very applied… if I'd gone and done a course on that six months ago, I probably wouldn't have recalled enough to apply it.”* (Chris-Consumer-FG-12mths)

Teaching the consumers new skills such as data analysis, was advantageous in crystallising the student’s learning. Co-learning was reinforced and became streamlined across different studies through repeated use of key papers and research skills. It was agreed that it was desirable and natural that all team members gained research skills and new knowledge. As described by Pat, *“…with that learning and development comes an insight which changes the lens through which you look.”* (Pat-Academic-FG-18mths) However, the concept of long-term consumer partnerships creating ‘professionalised consumers’ who no longer had a lay perspective and therefore could not add value was discussed at length and rejected. The student remarked, *“I don't say to my supervisors—you've been working with me for two years and you know what's going on, so… I'll get two new ones so they can have a fresh perspective.”* (Regi-Student- FG-18mths)

The academics were transparent about their learning which reinforced the co-learning and power sharing ethos. *“I know that I'm learning stuff as well… the stuff that we're reading, but also in learning about this process of truly trying to engage consumers in the research process… that it's a little bit slower, but actually, you get a lot more out of it in the end.”* (Sam-Academic-FG-12mths)

### Sub-theme: mutually beneficial and rewarding in the real world

There were benefits and rewards for the team members personally, for the research process and outcomes, and for driving change in consumer partnerships as time progressed. For example, Alex discussed personal benefits, *“…as I get physically, you know, less mobile, it's a thread that keeps going… enjoyable and stimulating.”* (Alex-Consumer-FG-24mths) Another consumer, Chris, talked about being retired and having a sense of purpose, *“…it's a wonderful feeling to be engaged in something that's ongoing and something that's informative and interesting and helpful.”* (Chris-Consumer-IV-6mths) Development of new skills such as in information technology, data analysis, CCI, and conference presentations were a source of pride for all. Additionally, working with the consumers reduced the isolation typically felt by doctoral candidates and prevented procrastination due to enhanced accountability. The student commented, *“… I don't want to let, particularly you… consumers down. I want to keep things going… I don't want you to be hanging around not feeling like anything's happening… we push each other along.”* (Regi-Student- FG-24mth) An academic also commented, *“…because you [the student] have a group that you're answerable to, you can't actually procrastinate too much and put things off, so you then actually prioritise that….”* (Pat-Academic-FG-24mths) The academics benefited through their use of the CCI as accreditation evidence and discussed how their learnings influenced other research and partnerships as opportunities arose during the 33-months. They also formed new networks for their consumer partnering work, *“…we [university department] need some good consumer reps to be involved in a few things, and I'm thinking… oh, here's two people who are ready to go… with lots to offer.”* (Sam-Academic- FG-30mths)

The consumers added enormous value to research planning, rigour, and dissemination through their many ‘why’ questions which tested tacit student and academic assumptions across all PhD studies. For example, deep discussion of the definition and rationale of quality improvement in healthcare generated many new search terms for a scoping review. Additionally, unique consumer perspectives improved data collection methods and interpretation with the student gaining an enhanced awareness of the limitations of her *“reductionist and positivist roots.”* (Regi-Student-Reflection- FN-Mtg5-5mths)

The consumers’ real-world advocacy, and implementation focus were an advantage in informing the ‘so what’ of each study which was immensely helpful in generating discussion points for manuscripts and presentations. Furthermore, tangible outputs such as the capability framework, consumer newsletter pieces, presentations, and publications were celebrated, creating energy in the long doctoral journey. A consumer reported, *“The knowledge translation wins provided me with extra energy and enthusiasm. We are kicking goals!”* (Consumer-Log-12mths) Co-authorship of peer reviewed papers was particularly gratifying. *“I personally made a huge contribution, I felt, to those themes, and I was quite chuffed… to see them there in print.”* (Chris-Consumer-FG-12mths) Additionally, as the team reputation grew, so too did opportunities to collaborate on external studies, co-produce educational videos, and consult on and lead health service CCI activities and policy.

### Sub-theme: a journey together with a beginning, middle, and end

The concept of the team being on a journey was raised many times throughout the 33 months as was a sense of a long-term partnership. As Chris said, *“I'm committed to this, and I want to see it through to the end.”* (Chris-Consumer- FG-18mths) Initially the CCI focus was on recruitment with selection criteria and detailed discussion about mutual expectations being core to maximising a good team fit. Orientation of recruited consumers then followed with an icebreaker at the first meeting leading to warmth and in-jokes. As Sam, an academic commented, “*We're working on us working together as a team… getting to know each other, building relationships, navigating relationships.”* (Sam-Academic- IV-6mths) However, just as teamwork was beginning to consolidate, COVID-19 restrictions commenced, necessitating online meetings which whilst not one consumer’s preference, later became necessary for another due to health and mobility issues. At first, the student frequently felt like *“the peacemaker and negotiator”* during discussions, but this eased as people became attuned to each other’s preferences, communication styles, and priorities. (Regi-Student-FN-Mtg17-14mths)

Building on experiences over the longer-term enhanced everyone’s confidence and the consumer impact. One consumer moved from being *“all at sea”* to *“Well, I'm a researcher now, so I'm influencing it lots (laughter).”* (Chris-Consumer-FN-Mtg1-1mth;FG-18mths) Additionally, there was an increasing sense of working as peers which reinforced equitable collaboration. An academic said, *“You [consumers] got a place on the team… because of your consumer perspective… we've moved beyond that and you're just two other members of the research team who have your perspective….”* (Sam-Academic-FG-18mths) Given it was a long-term and close partnership, consent to disclose personal information within and outside the team, and healthy personal boundaries were frequently discussed to address privacy and consent. Alex reflected, *“…you might find that you think of each other as friends, but we need to keep that separateness.”* (Alex-Consumer-FN-Mtg12-10mths)

A deliberate and ethical approach to closure of the partnership commenced nine months before completion of data collection of this ethnographic study, although a further three to five months of PhD-related collaboration was anticipated. An academic noted the discomfort associated with these discussions regarding closure of the partnership, *“Planning final analyses and PhD completion is perhaps more confronting than earlier phases.”* (Academic-Log-30mths) A proactive and supportive approach to concluding the rewarding and enjoyable partnership enabled preparation for the end and planning of future CCI work. Initially Alex disclosed that, *“I am going to be sad when our meetings finish. So, I guess I need to get my mind around that.”* (Alex-Consumer-FN-Mtg33-28mths) Several months later she discussed a new research collaboration she was considering and noted, *“I could fill that space with something once [student’s name] stuff is finished… it all interconnects and is a next step for me.”* (Alex-Consumer-FN-Mtg37-32mths)

## Discussion

This ethnographic study provides a novel, longitudinal, and in-depth evaluation of consumer partnerships in a PhD from the perspective of the consumer co-researchers, student, and academic supervisors. It demonstrates that due to doctoral research focussing on learning, growth, and supervision, a rich ethos for research and knowledge co-production was reinforced. Careful attention to building and maintaining strong, collegial relationships, and equalising power dynamics was essential [8, 9, 32]. The growth experienced went far beyond enhanced knowledge and confidence regarding research and CCI processes and incorporated substantial relational growth for future collaborations within and outside the team [8]. However, being a PhD, the student was required to lead which limited consumer leadership but also seemed to protect them from the inevitable frustrations and challenges that occurred. Emphasising that a doctoral student is required to drive the research during consumer recruitment for CCI in PhDs is therefore recommended to clarify expectations and roles from the beginning as is best practice [23, 33]. However, opportunities for consumer leadership such as developing new networks for recruitment and knowledge dissemination, and for activities such as qualitative theme naming should also be embraced and encouraged.

### Benefits of a co-production partnership in a PhD

Consistent with the literature, whilst considerable time and effort was required from all partners, there were many benefits to research outcomes including improved research accountability, quality, and relevance across the research cycle [9, 17, 32]. Additionally, the emphasis on authentic co-production, rather than consultation, avoided the risks of tokenism and instrumentalization of CCI [8, 13, 34]. Dissemination and translation of PhD findings was a large focus and provided substantial reward and recognition for the consumers. Co-production of knowledge translation ensured inclusion of consumer perspectives beyond traditional academic publications as recommended for enhanced implementation of research findings [15, 34]. Given journals are increasingly requiring a CCI statement [35], partnerships in a PhD may also be advantageous for publication success. Similar to CCI in other PhDs, the student and consumers reported enhanced well-being from the partnership, including reduced student isolation [20], and increased consumer self-esteem due to skill development and contributing to important research that addressed an identified need [15, 19]. For the consumers, a sense of purpose, being valued, altruism, and mental stimulation were additional positive outcomes described which concurred with other literature [7, 24, 36, 37]. A novel finding was that student procrastination may have been avoided. She was motivated to be highly productive so that the consumers had ongoing, enjoyable, and interesting work to do. The academic supervisors reported CCI skill development and formation of networks for future research.

### Strategies for authentic partnerships

Findings reinforced that monthly meetings kept the momentum going. In addition, a flexible but structured agenda with prioritised pre-reading, creative strategies to promote discussion and collaborative decision making, and dedicated time for team reflection provided direction without restricting collaboration. Having sufficient time for open dialogue and debate was essential to ensure that exploratory co-production processes were not stifled as discussed elsewhere [12, 34]. A small research team with at least two consumers promoted close, deep, and reciprocal relationships, equal contributions, and continuity [18]. Whilst implementation of a short term RAG for one study promoted diverse voices [29], it did not achieve the influence, depth, and richness of the long-term co-production partnership. Thus, advisory groups are not recommended as the sole CCI strategy in a PhD. The deliberate approach to ethical considerations is highly recommended. It provided a safe space to address potential ethical tensions including personal boundaries, privacy, power sharing, consumer burden, and confidentiality [13].

The mutual respect and trust set-up by the recruitment strategies, meeting processes, and longer-term relationships meant that disagreements and challenging discourse were encouraged, consistent with the principles of co-production [12, 33]. The findings here reinforced that genuine and proactive approaches to building trust and respect meant that the research team did not focus on internal disagreements in the evaluation but rather discussed external challenges faced as a cohesive team. Additionally, the 33-month partnership meant that considerable flexibility was required to accommodate changing circumstances both internal and external to the team [10]. Flexibility needs to be factored into PhD CCI. It was also essential to pay close attention to sensitively ending the partnership which is an area neglected in the literature and requires further research. The ongoing cycles of formal and informal evaluation and improvement promoted effective team work and consumer empowerment [38]. Thus, it is recommended that rigorous evaluation of CCI is factored into PhD planning and focusses on research impacts and outcomes for all partners [12].

### Capabilities, learning, and development

Similar to other CCI in PhD research, bespoke team co-learning developed and delivered by the student was core to ethical co-production [17, 19]. Responsive consumer capability development, with an emphasis on individual and team reflection which is supported by learning through experience is recommended. Additionally, like other PhD partnerships, consumer preference guided depth of research skill education and subsequent implementation which maximised consumer influence within their comfort zone [17, 19]. Supporting consumer learning created an initial time burden for the student but efficiencies were gained with repetition over several studies. Over time, the consumers developed many research capabilities. However, the concept of a ‘professionalised consumer’ who could no longer provide a unique perspective, was rejected as inconsistent with a commitment to power sharing. Further research regarding this topic is required [13] as is consumer leadership [39], particularly the balance between a doctoral student being required to demonstrate independence and originality, and opportunities to promote consumer leadership.

Additionally, in agreement with the literature, the student developed capabilities in group facilitation [15], relationship and communication management [10], tailored consumer support and training [18, 32], and working with partners from diverse backgrounds [10, 34, 40]. These capabilities are relevant to a future research career and should be a focus for PhD supervision, student reflection, and formal learning opportunities. Given the student was a highly experienced healthcare manager and CCI lead, the team questioned whether all PhD candidates could lead co-production as effectively. However, PhD candidates are increasingly mature learners with pre-existing work and life experiences [16], so this may be less of an issue than anticipated but requires further investigation.

### Implications for universities

Authors have called for a ‘radical disruption’ to the academy with a new vision and culture where academics and communities reciprocally seek and value each others’ knowledge [8, 9]. The research reported here has demonstrated that such partnerships can be highly successful in a PhD. However, the research team relied on links with a health service for consumer mandatory training, expense reimbursements, and access to consumers for recruitment. Whilst links with external organisations and stakeholders are advantageous to PhD students and universities [15], building long-term university co-production infrastructure is essential [14]. Lack of funding for consumer remuneration was frustrating and may have perpetuated the marginalisation of consumers from diverse backgrounds [32, 41]. Thus, it is essential that universities incentivise CCI, including dedicated funding for consumer remuneration for PhD studies [15, 18]. A recent publication provides valuable recommendations for reducing institutional barriers to consumer remuneration [41]. Normalising and expanding university innovations such as consumer academic appointments would also enhance parity of diverse perspectives [7, 39], and prevent issues such as lack of access to data and resources [19]. Formal supervision of PhD candidates by consumer leaders also warrants further investigation. The large time commitment, engaged presence, and openness to learning of the academic supervisors fostered respect for consumer voices. However, whilst this built critical academic capability in CCI [8] and development of much needed CCI champions [5], the right balance for sustainable academic supervisor engagement requires further examination.

### Limitations

This study occurred at an Australian university and thus it may not be generalisable to all settings and circumstances, especially as international universities may have a longer history of CCI in research with associated infrastructure. Additionally, all consumer co-researchers recruited were retired, had high literacy levels, and spoke English fluently which may have assisted in equalising the power dynamics. Findings indicated that consumers having sufficient time to devote to the research (mean 8.75 h per month) and being able to read complex research materials was considered essential for meaningful co-production. Hence, the feasibility of PhD candidate close collaboration with consumer co-researchers from more diverse backgrounds requires further research.

## Conclusions

This study has demonstrated that, with the focus of doctoral research on growth and learning across multiple studies and several years, co-production with consumers resulted in benefits for research outcomes, the student, consumers, and academic supervisors. Core ingredients for success included capable student leadership, recruitment of at least two consumers, and committed academic supervisor involvement. Regular structured meetings with a focus on ethical considerations, reflexive dialogue, collegial relationships, and practical tasks were also essential. Mutual support, ongoing commitment, and flexibility throughout the partnership journey with responsive co-learning beyond didactic teaching were also critical. A co-production paradigm may be essential to the success of modern universities with increasing calls for epistemic justice and more transparent academic contributions to communities. Thus, consumer and community involvement in doctoral studies equips students, academic supervisors, consumers, and universities for a future when co-production will be the norm.

## Supplementary Information

**Additional file 1.** GRIPP2* Reporting Checklist (Short Form).

## Data Availability

Due to the nature of the consent obtained, the raw data cannot be shared. Summary data which support the findings of this study are available from the corresponding author upon reasonable request.

## Abbreviations

CCI: Consumer and community involvement
HHS: Hospital and health service
IAP2: International Association of Public Participation
RAG: Research advisory group
