# Supplementary material for: ‘Learning and growing together’: exploring consumer partnerships in a PhD, an ethnographic study
Source: Res Involv Engagem. 2023 Mar 15;9:8. doi: 10.1186/s40900-023-00417-6 (PMC10014401; doi:10.1186/s40900-023-00417-6)
Supplement: Supplementary file 1 — Additional file 1. GRIPP2* Reporting Checklist (Short Form). [file 40900_2023_417_MOESM1_ESM.docx]

**File 2 supplementary materials: GRIPP2* Reporting Checklist (Short Form)**

| **Section & topic** | **Item** | **Section where reported and details** |
| --- | --- | --- |
| 1: Aim | Report the aim of Consumer and Community Involvement^ (CCI) in the study | The research aim specifically focusses on CCI in the Background section. The research questions also clearly focus on CCI in the Methods section. |
| 2: Methods | Provide a clear description of the methods used for CCI in the study | The Methods include a clear description of the consumer co-researcher recruitment, orientation, meeting processes, data analysis, interpretation, and write-up. |
| 3: Study results | Outcomes—Report the results of CCI in the study, including both positive and negative outcomes | The Findings include detailed information regarding the processes and outcomes of co-production with consumers, with the themes providing specifics about both the positives and negative outcomes. |
| 4: Discussion and  conclusions | Outcomes—Comment on the extent to which CCI influenced the study overall. Describe positive and negative effects | The Discussion focusses on co-production with consumers across the PhD program of research and all points are relevant to this specific study. Benefits and challenges of CCI are both included. |
| 5: Reflections / critical perspective | Comment critically on the study, reflecting on the things that went well and those that did not, so others can learn | Critical reflection regarding CCI during the PhD is included throughout the paper, particularly in the Findings. Recommendations for co-production in doctoral research are also included in the Discussion and Conclusions. |

^The GRIPP2 uses the term Patient and Public Involvement (PPI) which is the United Kingdom equivalent of CCI

* Staniszewska, S., J. Brett, Simera. S., K. Seers, C. Mockford, S. Goodlad, D. G. Altman, et al. 2017. "GRIPP2 reporting checklists: tools to improve reporting of patient and public involvement in research."  *Research Involvement and Engagement* 3 (13):11. doi: 10.1186/s40900-017-0062-2.
